# Clinical Significance of Erythroferrone and Bone Morphogenetic Protein-6 in Patients with Anemia in the Course of Inflammatory Bowel Disease

**DOI:** 10.3390/metabo13091006

**Published:** 2023-09-12

**Authors:** Małgorzata Woźniak, Anna Borkowska, Marta Jastrzębska, Marcin Sochal, Ewa Małecka-Wojciesko, Renata Talar-Wojnarowska

**Affiliations:** 1 Department of Digestive Tract Diseases, Medical University of Lodz, 90-419 Lodz, Poland; anna_bork@wp.pl (A.B.); ewa.malecka-panas@umed.lodz.pl (E.M.-W.); r-wojnarowska@wp.pl (R.T.-W.); 2 Department of Gastroenterology, Health Care Center, 26-200 Konskie, Poland; marta.bar@interia.pl; 3 Department of Sleep Medicine and Metabolic Disorders, Medical University of Lodz, 90-419 Lodz, Poland; sochalmar@gmail.com

**Keywords:** ferritin, erythroferrone, bone morphogenetic protein, anemia, inflammatory bowel disease

## Abstract

In recent years, a steady increase in the incidence of inflammatory bowel diseases (IBD) has been observed with anemia as their most common extraintestinal symptom. Erythroferrone and Bone Morphogenetic Protein 6 (BMP-6) are recently identified cytokines involved in the process of increased erythropoiesis in anemia of various pathomechanisms. The aim of this study was to analyze the concentration of erythroferrone and BMP-6 in IBD patients in relation to clinical and laboratory data. The study comprised 148 patients: 118 with IBD, including 73 (61.85%) diagnosed with anemia (42 with Crohn’s disease (CD) (66.7%) and 31 (56.4%) with ulcerative colitis (UC)) and 30 as a control group. The erythroferrone concentration was significantly higher in IBD patients with anemia (*p* = 0.009) and higher in UC patients both with and without anemia (*p* = 0.018), compared to the control group. In CD, no similar difference was observed between patients with and without anemia. Regarding BMP-6, higher levels were found in CD patients with anemia compared to the control group (*p* = 0.021). The positive correlation between BMP-6 and iron concentration in UC was also noticed. In conclusion, we confirm an increase in erythroferrone concentration in the entire group of IBD patients with anemia, while BMP-6 levels were higher only in anemic CD patients. Due to the clinical importance of anemia in IBD, this problem is worth further analysis and research projects.

## 1. Introduction

In recent years, a steady increase in the incidence of inflammatory bowel diseases (IBD) has been observed, both ulcerative colitis (UC) and Crohn’s disease (CD), with anemia as their most common extraintestinal symptom [1,2,3]. Due to multifactorial etiopathogenesis, differential diagnosis of anemia in patients with IBD exacerbation is still a significant clinical problem. The particular challenge is to differentiate anemia caused by iron deficiency from that of chronic diseases in which pathomechanism is not fully understood. The main cause of anemia in chronic diseases probably results from the adverse effect of pro-inflammatory cytokines on erythropoiesis. However, not all patients with IBD exacerbation manifest anemia in laboratory tests. Moreover, anemia may also be found in patients in clinical remission and without an increase in inflammatory markers [3,4].

The proper regulation of iron management in the human body is controlled by many mechanisms with involved cytokines and hormones. One such regulatory protein is hepcidin, which plays an important role in regulating iron absorption. Recent data from experimental studies also emphasize the significance of erythroferrone and bone morphogenetic proteins, especially Bone Morphogenetic Protein-6 (BMP-6). Hepcidin is a long-chain 25-amino acid peptide, rich in cysteine and containing four disulfide bonds [5]. Hepcidin, combined with ferroportin, a protein that controls the outflow of iron from the cell, causes its phosphorylation and internalization, and through it, iron retention in enterocytes and its removal, along with the process of intestinal epithelial exfoliation. It is known that with hepcidin deficiency, there is an increased intestinal absorption of iron and its increased release from macrophages [5,6,7].

On the other hand, erythroferrone and BMP-6 are recently identified proteins involved in the process of increased erythropoiesis in anemia of various pathomechanisms. Data on these proteins come mainly from experimental work in animal models. Erythroferrone, first described in 2014, is encoded by the ERFE gene on chromosome 2, comes from erythroid precursor cells, and its concentration increases in the case of iron deficiency [8]. Erythropoietin is involved in this process, affecting the expression of erythroferrone by activating the JAK2/STAT5 signaling pathway, which stimulates the process of erythropoiesis [8,9]. In experimental studies, the effect of erythroferrone on hepcidin was proven by administering it to healthy mice and observing a significant decrease in its concentration [9]. Also, in the group of tested animals with hemolytic anemia, an increase in the concentration of erythropoietin and erythroferrone was shown, accompanied by a decrease in the concentration of hepcidin [10]. One of the few clinical studies proved that erythroferrone could be used in the treatment of anemia in patients with chronic renal failure undergoing hemodialysis [11].

Erythroferrone is a regulator of the BMP-6 protein, belonging to the transforming growth factor β (TGF-β) family, mediating the regulation of hepcidin expression depending on the iron content in the body [12,13]. Bone morphogenetic proteins, in addition to an important role in the processes of neuro- and chondrogenesis, participate in the regulation of iron management in the body [13,14]. Currently, more than 20 BMP proteins are known, including BMP-6, consisting of more than forty ligands. BMP-6 specifically binds to the serine–threonine receptor on the surface of liver cells, causing the phosphorylation of proteins that transmit the signal to the cell nucleus and stimulating the transcription of the hepcidin gene [15,16]. Experimental studies show that erythroferrone reduces the concentration of BMP-6 protein in the liver, and hence reduces the concentration of hepcidin and increases the availability of iron [17]. BMP-6 may have a potentially beneficial role in the treatment of anemia caused by excess hepcidin, and the use of BMP-6 agonists is considered in the treatment of iron overload disorders [13].

There are no data on the assessment of the importance of erythroferrone and BMP-6 in IBD. According to the analysis of the available literature, these proteins have not been tested in this group of patients so far. The aim of the presented study is to analyze the concentration of erythroferrone and BMP-6 in IBD patients with concomitant anemia in relation to clinical and laboratory data in the study group.

## 2. Materials and Methods

All subsequent IBD patients hospitalized at the Department of Digestive Tract Diseases at the Medical University of Lodz between 2017 and 2019 were qualified for this prospective study. The study included 118 adult patients with IBD; in the analyzed group 73 (61.85%) were diagnosed with anemia, including 42 with CD (66.7%) and 31 (56.4%) with UC. After admission to our department from each patient with IBD, who had consented to participate in the study, an additional peripheral blood sample (1 mL) was collected during routine blood sampling for laboratory tests. Depending on the result of morphology, patients were qualified for the group with or without anemia. According to the definition by the World Health Organization (WHO), anemia is defined as HGB < 12 g/dL in non-pregnant women and <13 g/dL in men. Mild anemia is diagnosed with HGB > 11 g/dL, moderate with HGB 8–11 g/dL, and HGB < 8 g/dL is considered to be severe anemia [18]. The study excluded patients with a recent history of anemia treatment, including transfusion of red blood cell concentrate or supplementation with iron, folic acid, or vitamin B12 within 3 months before the current hospitalization. Exclusion criteria included other accompanying diseases that affected iron management, such as hemochromatosis, porphyria, or thalassemia, as well as bone marrow diseases including myelodysplastic syndrome. Moreover, patients with other chronic inflammatory diseases were also excluded from our study. The control group consisted of 30 healthy volunteers without anemia in laboratory tests and without chronic diseases, in whom the image of the colon was normal in a colonoscopy performed for other indications. Each patient signed a written consent to participate in the study. Approval from the Bioethics Committee of the Medical University of Lodz was obtained for the study (RNN/149/16/KE).

Each hospitalized patient from the study group, apart from routine laboratory tests, had 1 mL of peripheral blood collected to determine the concentration of hepcidin, erythroferrone, and BMP-6. The blood was centrifuged for 20 min in a centrifuge at a speed of 1000× *g* and then frozen for further determinations at −80 °C. BMP-6 and erythroferrone concentrations were determined using the ELISA enzyme immunoassay with kits from WuHan EIAab Science Co., (Wuhan, China) and the concentration of hepcidin with kits from R&D Systems. The minimum detected concentration for the tested parameters was, respectively, as follows: BMP-6—0.156 ng/mL; erythroferrone—0.156 ng/mL; and hepcidin—0.446 ng/mL.

In the statistical analysis, the results were expressed as mean +/− standard deviation. The assumption of the normal distribution of differences was verified with the use of the Shapiro–Wilk test. As the normality assumption was violated, the significance of differences was tested with the Mann–Whitney’s U test to compare two independent groups. Inter-group differences of categorical variables were analyzed using the chi-square test. Spearman analysis was used to determine the significance of correlation. *p*-values less than 0.05 were considered statistically significant. All statistical calculations were performed using the Statistica 13.1 program by StatSoft, Inc. (Cracow, Poland).

## 3. Results

During the analyzed time period, 118 IBD patients qualified for the study: 55 (46.61%) with UC and 63 (53.39%) with CD. There were no significant differences in gender in both UC and CD (*p* > 0.05). The average age of IBD patients was 42.5 years (38.7 years in patients with CD and 46.8 years in patients with UC) with no significant differences in gender (*p* > 0.05). The mean Crohn’s Disease Activity Index (CDAI) score was 335.06 ± 150.25. Anemia was found significantly more often in patients with CD—42 (66.7%)—compared to 31 (56.4%) patients with UC (*p* = 0.033). The prevalence of anemia increased significantly with the severity of IBD and the extent of inflammatory changes in the endoscopic examination, both in patients with CD and UC. The clinical data of analyzed patients along with the disease location and endoscopic results were presented in our previous work [19]. 

**Figure 1:**
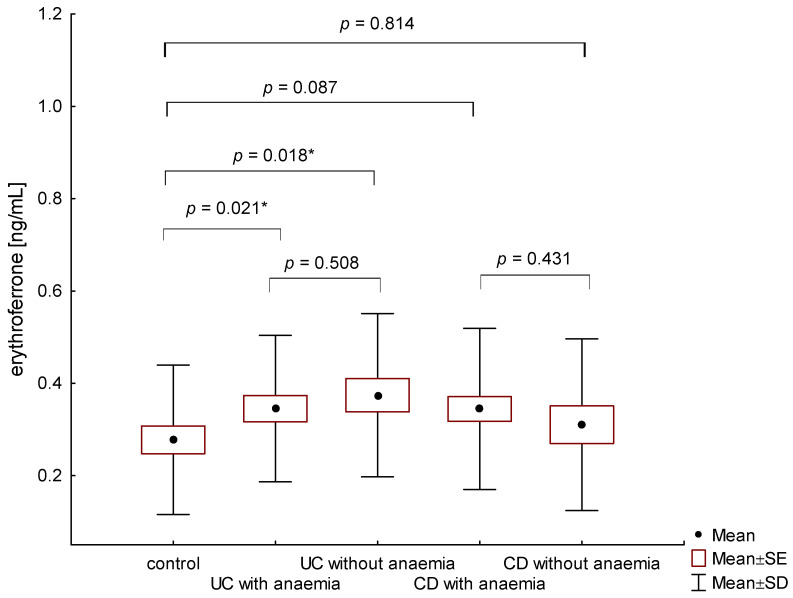
The concentration of erythroferrone in patients with UC and CD with and without anemia in relation to the control group (*—*p* < 0.05).

The average concentration of erythroferrone in the group of anemic IBD patients was statistically significantly higher than in the control group and amounted to 0.345 ± 0.167 ng/mL and 0.275 ± 0.160 ng/mL, (*p* = 0.009), respectively. Patients with UC, both with and without anemia, had higher erythroferrone concentrations compared to the control group (respectively, 0.345 ± 0.159 ng/mL vs. 0.275 ± 0.160 ng/mL, *p* = 0.021 and 0.374 ± 0.177 ng/mL vs. 0.275 ± 0.160 ng/mL, *p* = 0.018) (Figure 1). However, there were no significant differences between erythroferrone levels in patients with UC compared to CD, regardless of the presence of anemia (*p* = 0.759). In the group of patients with CD, no statistically significant difference was found between patients with and without diagnosed anemia (0.344 ± 0.174 ng/mL vs. 0.310 ± 0.186 ng/mL, *p* = 0.431) (Figure 1).

**Figure 2:**
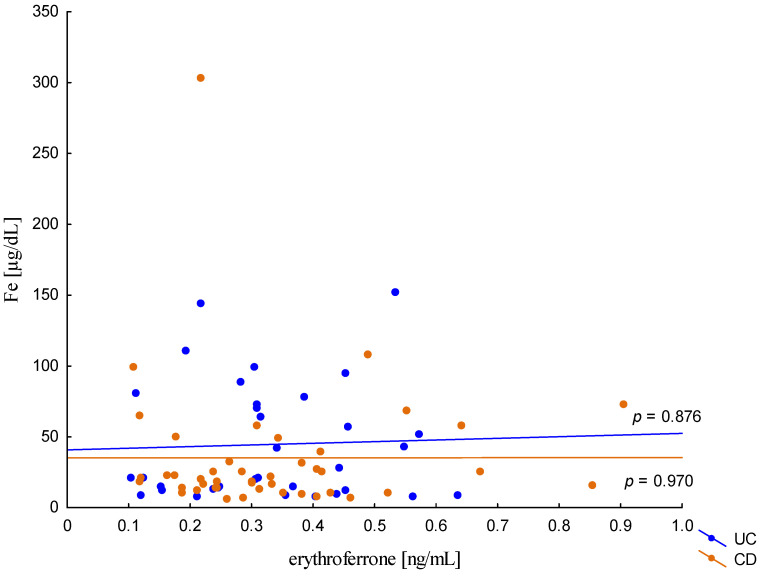
The correlation of iron concentration against erythroferrone concentration in patients with UC and CD.

**Figure 3:**
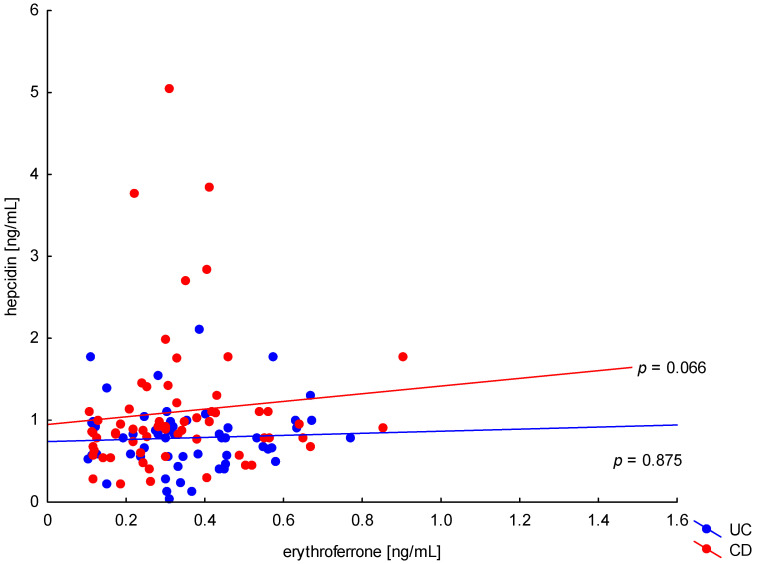
The correlation of hepcidin concentration against erythroferrone concentration in patients with UC and CD.

**Figure 4:**
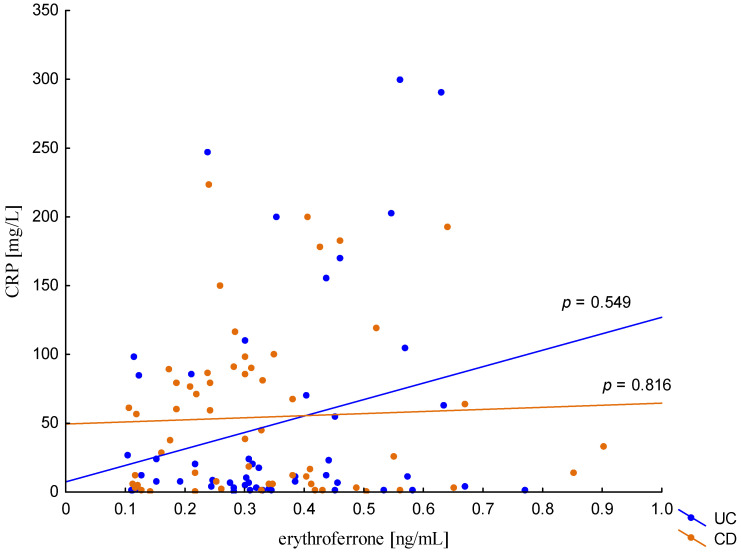
The correlation of CRP concentration against erythroferrone concentration in patients with UC and CD.

The relationship between the concentration of erythroferrone and the concentration of iron or hepcidin, reported in experimental studies, was not confirmed (Figure 2 and Figure 3). The average concentration of hepcidin in anemic UC patients was 0.656 ± 0.321 ng/mL and was significantly lower compared to UC patients without anemia (0.945 ± 0.449 ng/mL; *p* = 0.042) and the control group (1.016 ± 0.400 ng/mL; *p* < 0,001). Comparing only the groups with anemia, it was shown that the concentration of hepcidin in UC patients was significantly lower than in CD patients (0.656 ± 0.321 ng/mL vs. 1.16 ± 0.946 ng/mL, *p* = 0.004). In our study, no statistical differences in hepcidin levels were found, depending on the severity of the disease, CRP concentration, or the location of changes in both UC and CD (*p* > 0.05). Similarly, there was no relationship between erythroferrone and CRP levels in both UC and CD patients, which is consistent with previous reports (Figure 4).

**Figure 5:**
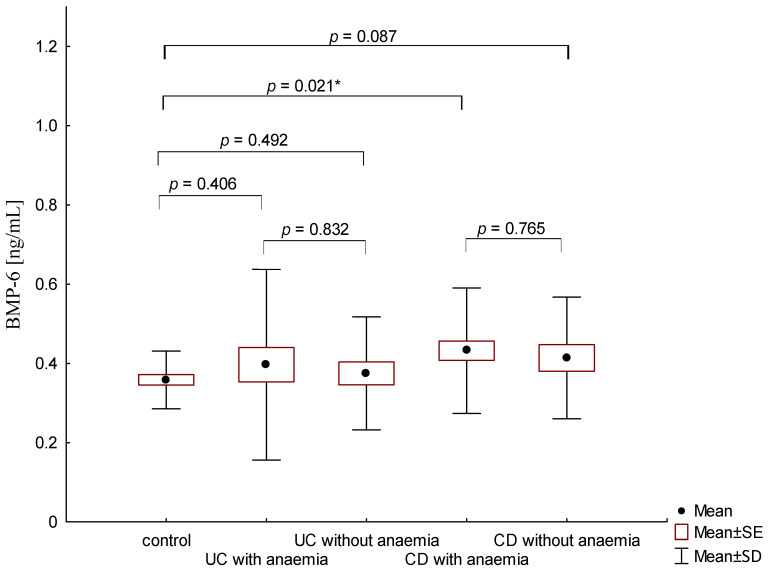
The concentration of BMP-6 in patients with UC and CD with and without anemia in relation to the control group (*—*p* < 0.05).

**Figure 6:**
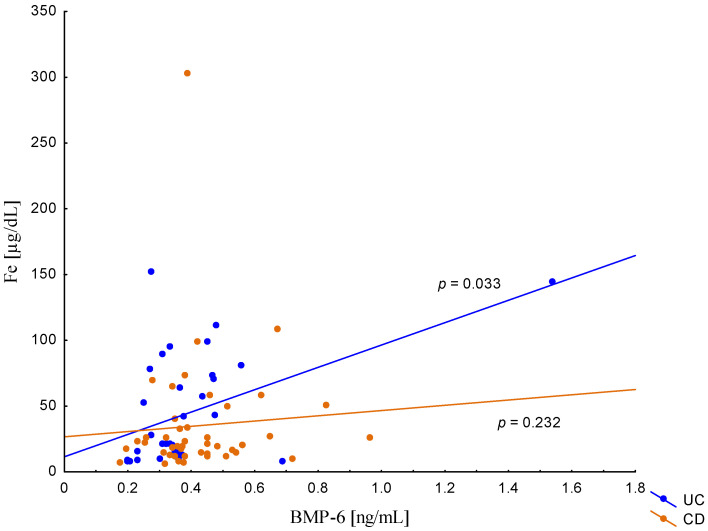
The correlation of iron concentration against BMP-6 concentration in patients with UC and CD.

**Figure 7:**
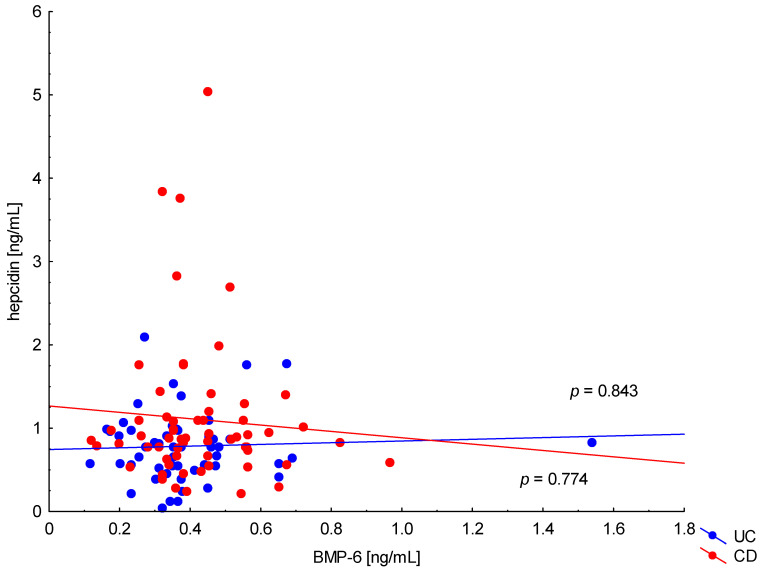
The correlation of hepcidin concentration against BMP-6 concentration in patients with UC and CD.

**Figure 8:**
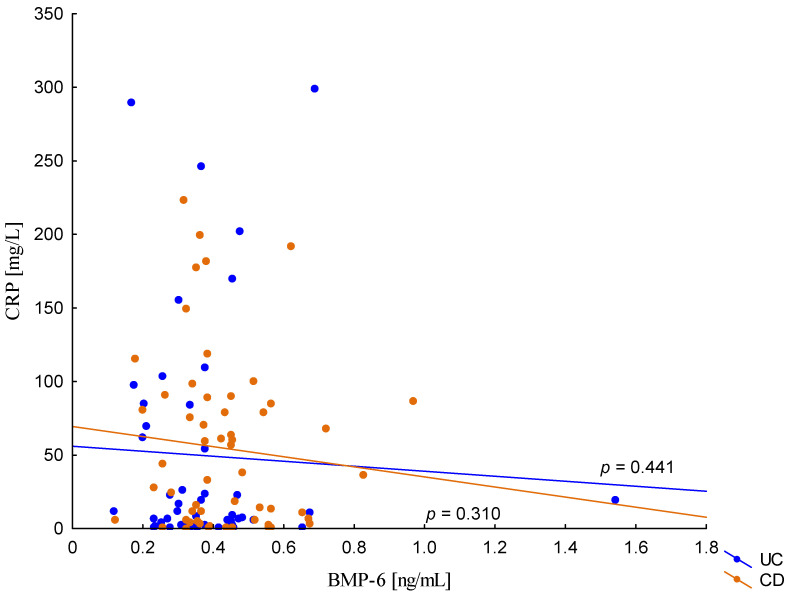
The correlation of CRP concentration against BMP-6 concentration in patients with UC and CD.

On the other hand, when analyzing the differences in BMP-6 concentration in the studied groups of patients, significantly higher BMP-6 levels were found in patients with CD with concomitant anemia compared to the control group (0.432 ± 0.158 ng/mL vs. 0.359 ± 0.072 ng/mL, *p* = 0.021). Such a difference was not found in CD patients without confirmed anemia (*p* > 0.05). Similarly, no significant differences in the BMP-6 concentrations range were observed in UC patients (Figure 5). However, when analyzing the relationship between the concentrations of BMP-6 and iron, a positive correlation between these parameters was revealed in patients with UC (*p* = 0.033). However, such a relationship was not observed in CD patients (*p* = 0.232) (Figure 6). There was also no statistically significant relationship between BMP-6 and hepcidin, despite such reports from experimental studies (*p* > 0.05) (Figure 7). As in the case of erythroferrone, no significant relationship was observed between BMP-6 concentration and the intensity of inflammation expressed by CRP concentration (Figure 8).

## 4. Discussion

Although anemia is the most common extraintestinal manifestation of IBD, its complex pathomechanisms still raise many questions. Most of the studies published so far confirm the relationship between the activity of IBD and the presence of anemia, but it is known that it may also affect patients in remission [3,20]. Due to the chronic inflammation in IBD, the insufficient role of ferritin, an acute phase protein, in the correct diagnosis of iron deficiency anemia is emphasized [21]. Due to the insufficient sensitivity of other markers in the differential diagnosis of anemia in IBD, high expectations were associated with the determination of hepcidin, as well as new laboratory parameters including erythroferrone and BMP-6 levels.

The present study showed that the hepcidin concentration was significantly lower in patients with UC and anemia compared to the control group, while no such relationship was observed in patients with CD. It is known that the decreased hepcidin level is associated with increased intestinal absorption of iron and its increased release from macrophages. Therefore, a low hepcidin level is a beneficial phenomenon in patients with UC and with anemia caused by iron deficiency. Due to the role of hepcidin in the regulation of iron metabolism, it is believed that inhibition of its expression may be beneficial in the treatment of anemia of complex etiology in the course of IBD [22]. The possible role of hepcidin as an indicator of the body’s response to oral iron supplementation is also highlighted. Bergman et al. showed that in patients with iron deficiency anemia and elevated hepcidin levels, oral iron supplementation was ineffective. However, these patients responded well to intravenous iron preparations [23].

According to the results of experimental studies, erythroferrone reduces the concentration of hepcidin, and hence, increases the availability of iron and increases the production of red blood cells in the erythropoiesis process. In our study, elevated levels of erythroferrone were observed in the entire group of anemic IBD patients, both in the course of CD and UC, which supports the involvement of this cytokine in the process of erythropoiesis. It is well known that the main organ responsible for the production of erythroferrone is the bone marrow, and the concentration of this protein significantly increases after bloodletting in mice [8,9]. To our knowledge, no studies evaluating the concentration of erythroferrone in patients with IBD have been conducted so far, and all data on this cytokine come from experimental studies. Based on previous reports, it is assumed that erythroferrone may play an important role in the regulation of hepcidin concentration and iron metabolism, and may even be a potential therapeutic target in some diseases accompanied by anemia [24]. In the presented study, however, we did not confirm the relationship between erythroferrone and iron or hepcidin levels in the analyzed groups of patients. As mentioned, previous analyses reporting such a relationship were carried out only on IBD animal models, which requires further clinical evaluation.

American researchers showed a significant decrease in hepcidin concentration after administration of recombinant erythroferrone to healthy mice [9]. Similar findings were made by a team of researchers in China who analyzed erythropoietin and erythroferrone concentrations in mice with laboratory-induced hemolytic anemia. It was observed that in the group of tested laboratory animals, there was a significant increase in serum erythropoietin concentration, with increased expression of erythroferrone in the bone marrow and spleen, with a simultaneous decrease in hepcidin expression [10]. On the other hand, an increase in erythroferrone concentration in the case of anemia resulting from defects in hemoglobin synthesis resulted in an ineffective increase in iron concentration. It was shown that an additional increase in the concentration of erythroferrone in mice with β-thalassemia increased the absorption of iron, but it was not used in the erythropoiesis process due to a defect in hemoglobin production and was consequently deposited in tissues [25].

The mechanism of erythroferrone action, its significance, and potential therapeutic possibilities require further research, especially in the clinical aspect. One of the few studies conducted in patients with chronic renal failure undergoing hemodialysis assessed whether erythroferrone could be used in the treatment of anemia in these patients. It has been shown that the increase in the concentration of erythroferrone, and thus the suppression of hepcidin and the increase in the availability of iron resources, may be induced by darbepoetin alfa and a continuous activator of the erythropoietin receptor [11]. This mechanism requires further research, but it could potentially be used in the treatment of anemia in dialyzed patients.

On the other hand, the analysis of the BMP-6 concentration showed that it is significantly higher in patients with CD and anemia compared to the control group. Such a relationship was not observed in patients with UC. To our knowledge, no studies on the clinical significance of BMP-6 in patients with IBD have been conducted so far, and all reports come from experimental studies. It is known that in conditions of elevated iron concentration in the serum, BMP-6 undergoes increased transcription only in the liver, in particular in the endothelial cells of the hepatic sinuses [16], while in other tissues it remains unchanged [26]. The BMP-6 protein, along with hemojuvelin (HJV) and transferrin, is one of the proteins involved in the signaling pathway that stimulates or inhibits hepcidin synthesis [13,27]. Despite earlier reports from experimental studies, the analyzed study did not confirm the relationship between BMP-6 and hepcidin concentrations in patients with IBD. In one of the experimental studies conducted on the effect of BMP-6 on the regulatory mechanisms of iron concentration in mice, it was proved that the administration of BMP-6 causes an increase in hepcidin concentration and, consequently, a decrease in serum iron concentration. The influence of both endogenous and exogenous BMP-6 on hepcidin expression and iron metabolism was assessed. The tested animals were injected once with a preparation containing BMP-6 at doses of 250 and 1000 µg/kg, obtaining a significant increase in hepcidin expression in the liver, and a dose-dependent decrease in iron concentration and transferrin saturation in serum [13]. The opposite mechanism was also analyzed by administering anti-BMP-6 antibodies at a dose of 10mg/kg to mice for 3 days, which significantly reduced hepcidin expression in the liver and increased iron concentration and transferrin saturation in the blood serum compared to animals from the control group. Additionally, mice lacking the gene responsible for the synthesis of BMP-6 were tested and showed an almost tenfold reduction in the expression of hepcidin in the liver and an increased concentration of iron in the serum at transferrin saturation approaching 100% [13]. Differences between the results of experimental and clinical studies require further research on various disease entities with anemia, including IBD.

The use of BMP-6 antagonists may have a beneficial effect in the treatment of anemia caused by excess hepcidin. Theurl et al. evaluated the effectiveness of BMP inhibitors in the treatment of anemia resulting from chronic inflammation. In rats with anemia, a dorsomorphin derivative or a soluble form of HJV were used as inhibitors of the BMP-SMAD pathway, resulting in an increase in the mobilization of iron from body reserves and an increase in erythropoiesis. However, the use of the dorsomorphin derivative is limited due to its side effects caused by non-selective action [28]. In another study, conducted in mice with induced colitis, the effect of inhibition of the BMP-SMAD axis on the concentration of hepcidin was assessed. Mice were given one of three anti-BMP agents: recombinant HJV protein, a small molecule BMP signal transduction inhibitor, or an anti-BMP-6 antibody. Blocking hepcidin expression with one of these three inhibitors has been shown to significantly increase serum iron concentrations in all cases, and furthermore, to reduce the severity of intestinal inflammation. This mechanism may be helpful in the treatment of anemia associated with inflammation and increased concentration of hepcidin [27].

In the presented study, no statistically significant relationship was observed between the concentration of the tested laboratory parameters, both erythroferrone and BMP-6 and the inflammation expressed by the CRP concentration. In published experimental studies, no changes in erythroferrone concentration were observed under the influence of generalized inflammation [8,9,10]. This is potentially significant for the differential diagnosis of anemia in both remission and exacerbation of IBD. However, the influence of hypoxia of erythroferrone concentration is still unclear. Emrich et al. showed in their study that increased erythroferrone levels were found in subjects exposed to high-altitude hypoxia [29]. On the other hand, other studies present no influence of hypoxia on erythroferrone [8,9,10].

In our work, no statistically significant relationship was observed between the concentrations of hepcidin and CRP. The influence of inflammation on hepcidin levels was shown in many other published studies [5,6,23]. According to some researchers, the increase in hepcidin concentration in response to inflammation is part of the body’s non-specific defense strategy, as the availability of iron is also limited for microbial cells. The possible increase in the concentration of hepcidin in chronic inflammatory diseases additionally intensifies iron metabolism disorders and is important, especially in the context of the role of hepcidin in the pathomechanism of anemia in IBD [5,23].

## 5. Conclusions

In conclusion, the presented results confirm an increase in erythroferrone concentration in the entire group of IBD patients with anemia, while BMP-6 levels were significantly higher only in patients with CD and anemia compared to the control group. The obtained results support the participation of the studied cytokines in the erythropoiesis process, but they require further clinical verification. Due to the frequency of anemia in IBD and its clinical importance, this problem is worth further analysis and research projects, concerning both erythroferrone and BMP-6.

## Data Availability

The data presented in this study are available upon request from the corresponding author. Data is not publicly available due to privacy or ethical restrictions.
